# Transcriptome Analysis Identifies Immune Markers Related to Visceral Leishmaniasis Establishment in the Experimental Model of BALB/c Mice

**DOI:** 10.3389/fimmu.2019.02749

**Published:** 2019-11-26

**Authors:** Maria Agallou, Evita Athanasiou, Olga Kammona, Spyros Tastsoglou, Artemis G. Hatzigeorgiou, Costas Kiparissides, Evdokia Karagouni

**Affiliations:** ^1^Parasite Immunology Group, Department of Microbiology, Hellenic Pasteur Institute, Athens, Greece; ^2^Chemical Process & Energy Resources Institute, Centre for Research and Technology Hellas, Thessaloniki, Greece; ^3^DIANA-Lab, Department of Electrical & Computer Engineering, University of Thessaly, Volos, Greece; ^4^DIANA-Lab, Hellenic Pasteur Institute, Athens, Greece; ^5^Department of Chemical Engineering, Aristotle University of Thessaloniki, Thessaloniki, Greece

**Keywords:** vaccines, visceral leishmaniasis, transcriptome analysis, neutrophils, disease establishment

## Abstract

Visceral leishmaniasis (VL) caused by *Leishmania donovani* and *L*. *infantum* is a potentially fatal disease. To date there are no registered vaccines for disease prevention despite the fact that several vaccines are in preclinical development. Thus, new strategies are needed to improve vaccine efficacy based on a better understanding of the mechanisms mediating protective immunity and mechanisms of host immune responses subversion by immunopathogenic components of *Leishmania*. We found that mice vaccinated with CPA_162−189_-loaded p8-PLGA nanoparticles, an experimental nanovaccine, induced the differentiation of antigen-specific CD8^+^ T cells in spleen compared to control mice, characterized by increased dynamics of proliferation and high amounts of IFN-γ production after *ex vivo* re-stimulation with CPA_162−189_ antigen. Vaccination with CPA_162−189_-loaded p8-PLGA nanoparticles resulted in about 80% lower parasite load in spleen and liver at 4 weeks after challenge with *L. infantum* promastigotes as compared to control mice. However, 16 weeks after infection the parasite load in spleen was comparable in both mouse groups. Decreased protection levels in vaccinated mice were followed by up-regulation of the anti-inflammatory IL-10 production although at lower levels in comparison to control mice. Microarray analysis in spleen tissue at 4 weeks post challenge revealed different immune-related profiles among the two groups. Specifically, vaccinated mice were characterized by similar profile to naïve mice. On the other hand, the transcriptome of the non-vaccinated mice was dominated by increased expression of genes related to interferon type I, granulocyte chemotaxis, and immune cells suppression. This profile was significantly enriched at 16 weeks post challenge, a time-point which is relative to disease establishment, and was common for both groups, further suggesting that type I signaling and granulocyte influx has a significant role in disease establishment, pathogenesis and eventually in decreased vaccine efficacy for stimulating long-term protection. Overall, we put a spotlight on host immune networks during active VL as potential targets to improve and design more effective vaccines against disease.

## Introduction

Leishmaniasis is a vector-borne disease caused by parasites of the genus *Leishmania* and is transmitted by the bite of female sand-flies to mammalian hosts. It is a poverty-related disease with three main clinical forms, visceral, cutaneous, and mucocutaneous leishmaniasis. Visceral leishmaniasis (VL) is the most severe, systemic form of the disease that is usually fatal if left untreated. Despite the fact that the global incidence of VL has decreased substantially in the past decade as a result of better treatment and vector control, in east Africa the case numbers continue to be sustained (1). For decades, VL has been treated by pentavalent antimonial monotherapy. However, the increasing numbers of non-responsive patients in India have led to increased dosage recommendations with severe toxic side-effects (2). Thus, the development of prophylactic vaccines against leishmaniasis is an urgent need for the control of infection. However, to date there is no registered vaccine for the prevention of human VL. Several candidates that incorporate a range of antigens are in pre-clinical development, but only few of them are in clinical studies (3, 4).

The quest for the missing vaccine for human VL requires the understanding of the infectious process which is still not clearly defined. Experimental models of *Leishmania* infection play a major role in understanding parasite biology, host-pathogen interaction, disease pathogenesis, and parasite transmission. The use of inbred mice was indispensable for the establishment of the T_H_1/T_H_2 paradigm that elegantly explains the axis of resistance vs. susceptibility to cutaneous disease (5), which however does not apply fully in VL (6). In the experimental model of VL, control of parasite replication requires an early and strong T_H_1 response with production of IL-12 and IFN-γ. However, the parasite seems to impair host cell function through CD8^+^ and CD4^+^ T cell exhaustion and differentiation of double-producing IFN-γ and IL-10 Tr1 cells (7). Moreover, it has been shown that immune suppression in spleen during chronic infection is related to the induced structural alterations in spleen tissue architecture leading to splenomegaly (8). Therefore, the mechanisms leading to immune cell suppression and down-regulation of protective immune responses should be fully understood.

In the present study, we tried to shed light into the immune mechanisms related to infection or protective immune responses against VL using an experimental PLGA nanovaccine as a vaccine model. For this reason, we encapsulated into PLGA nanoparticles a 30-mer multi-epitope peptide consisting of multiple overlapping MHC class I and II epitopes obtained from the sequence of *L*. *infantum* Cysteine Protease A (CPA) that are able to induce humoral and cellular immune responses (9). Moreover, PLGA nanovaccines were surface modified with an octapeptide mimicking TNFα for efficient targeting of TNFRII on the surface of dendritic cells (DCs), namely p8, able to elicit protective anti-*Leishmania* CD8^+^ T cell responses (10). Accordingly, in the present study we found that subcutaneous vaccination of CPA_160−189_-loaded p8-PLGA nanoparticles induced antigen-specific CD8^+^ T lymphocytes in the spleen of BALB/c mice. Vaccination provided early but transient protection to *L*. *infantum* infection and was able to restrain the inflammatory response detected in the non-vaccinated mice as revealed by transcriptome analysis in splenic tissues of infected mice. Thus, we were able to identify molecules and pathways that related to VL establishment in the spleen of BALB/c mice. These molecules can be used in novel immunomodulatory interventions with ultimate goal the development of substantially enhanced anti-leishmanial vaccines.

## Materials and Methods

### Preparation of CPA_160−189_-Loaded p8-PLGA Nanoparticles

Poly (lactide-*co*-glycolide) (PLGA) 75:25 (Resomer RG752H, MW: 4–15 kDa), polyvinyl alcohol (PVA) (MW: 30–70 kSa, 87–90% hydrolyzed), phosphate-buffered saline (PBS; 10 × , pH 7.4), N-(3-Dimethylaminopropyl)-N'-ethylcarbodiimide hydrochloride (EDC), and N-hydroxysuccinimide 98% (NHS) were purchased from Sigma-Aldrich (Vienna, Austria). All chemicals used in this study were of analytical grade and commercially available. CPA_160−189_ peptide obtained from the sequence of *L. infantum* CPA protein (GenBank Acc. No.: CAM67356) was synthesized by GeneCust (Labbx, Dudenange, Luxenbourg) with purity ≥95% and a synthetic octapeptide, namely p8 (CTTYQGKL) that mimics the TNFα-docking region with the TNFRII, was synthesized by JPT (Berlin, Germany).

For the preparation of PLGA nanoparticles containing the CPA_160−189_ peptide, 0.3 mL of the peptide solution in PBS (6.6 mg/mL) was added into 3 mL of a PLGA/chloroform solution at a final concentration of 30 mg/mL. Then, PLGA nanoparticles were surface-modified with p8 peptide via a two-step carbodiimide method, as previously described (11, 12). Briefly, 1.5 mL of 7 wt% EDC solution and 1.5 mL of a 0.3 wt% NHS solution both prepared in 20 mM HEPES/NaOH buffer containing 1% (v/v) Pluronic F-68 at pH 7.0 were added into 1 mL of PLGA nanoparticles (empty or loaded with CPA_160−189_ peptide) dispersion in the same buffer at a final concentration of 20 mg/mL, in order to activate the PLGA carboxyl groups (13). The mixture was stirred end-over-end for 2 h at room temperature. The residual reagents were removed by centrifugation at 13,860 × g for 10 min at 25°C. Subsequently, 0.3 mL of 0.1% (w/v) p8 solution in 20 mM HEPES/NaOH buffer containing 1% (v/v) Pluronic F-68 at pH 7 were added to the PLGA NPs dispersion, and the mixture was incubated for 18 h at room temperature. To saturate free-coupling sites, 0.5 mL of 20% (w/v) glycine in 20 mM HEPES/NaOH containing 1% (v/v) Pluronic F-68 at pH 7.0 was added and incubated end-over-end for 1 h at 25°C. The PLGA nanoparticles were subsequently purified by means of two successive centrifugation-redispersion cycles at 13,860 × g for 10 min at 25°C in the same buffer. Finally, to prepare p8-PLGA nanoparticles, the CPA_160−189_ peptide solution was replaced with 0.3 mL of PBS. The obtained PLGA nanoparticles were stored at 4°C for analysis and further use.

### Characterization of PLGA Nanoparticles

The average particle diameter of the PLGA nanoparticles was determined by photon correlation spectroscopy and their zeta potential by aqueous electrophoresis measurements (Nano ZS90; Malvern Instruments Ltd., Malvern, UK). The measurements were performed with aqueous dispersions of PLGA nanoparticles prior to lyophilization.

To determine the loading efficiency of CPA_160−189_ and p8 peptide in PLGA nanoparticles, ~2.5 mg of nanoparticles were dissolved in 0.25 mL DMSO for 1 h following a further dissolution in 1.25 mL of 0.05 N NaOH and 0.5% (v/v) SDS for 3 h at 25°C. The amount of CPA_160−189_ was determined by MicroBCA Protein assay kit (Thermo Scientific) following manufacturer's instructions at 562 nm using a microplate reader (EL808IU-PC; Biotek Instruments Inc, Winooski VT, USA). Empty PLGA nanoparticles were used as controls. The amount of p8 that was conjugated on PLGA nanoparticles' surface was determined with a UV-Vis spectrophotometer (Lamda 35; PerkinElmer, Waltham, MA, USA) measuring fluorescence activity of FITC at 492 nm. The CPA_160−189_ encapsulation efficiency (EE%) as well as p8 conjugation efficiency (CE%) were calculated by the ratio of CPA_160−189_ or p8 mass in/on PLGA nanoparticles over the total mass of CPA_160−189_ or p8 used. Peptide load (CPA_160−189_ or p8) (wt%) was calculated by the ratio of the encapsulated or conjugated mass of peptide over the total mass of the PLGA nanoparticles.

### Mice, Parasite Culture, and Preparation of Soluble *Leishmania* Antigen

Studies were performed with female 6–8 weeks old BALB/c mice reared in the pathogen-free animal care facility at Hellenic Pasteur Institute. They were housed in a climatically controlled room receiving a diet of commercial food pellets and water *ad libitum*. All efforts were made to minimize animal suffering.

*L. infantum* (MHOM/GR/2001/GH8) strain was cultured *in vitro* at 26°C in RPMI-1640 (Biochrom AG, Berlin, Germany) supplemented with 2 mM L-glutamine, 10 mM HEPES, 24 mM NaHCO_3_, 100 U/mL penicillin, 10 μg/mL streptomycin and 10% (v/v) heat-inactivated fetal bovine serum (Gibco, Paisley, UK) which will be referred as complete RPMI.

Soluble *Leishmania* antigen (SLA) was prepared according to a previously described protocol (14). Briefly, 1 × 10^9^ late-log phase promastigotes were washed thrice in PBS and disrupted by five repeated freezing/thawing cycles (liquid N_2_/37°C) following by 5 min incubation on ice. Partially lysed material was exposed for 30 s in a sonicator and then centrifuged for 30 min at 8,000 × g at 4°C. The supernatant containing the SLA was collected and was stored in aliquots at −80°C in aliquots until use.

### Organ Collection and Isolation of Immune Cells

To isolate bone marrow-derived cells for the differentiation of dendritic cells (DCs), femurs and tibiae of the mice were flushed with RPMI in a 100 mm Petri dish. Cells were strained through a 70 μm cell strainer, centrifuged and red-blood cells were lysed with ammonium chloride lysis solution (ACK; 0.15 M NH_4_Cl, 1 mM KHCO_3_, 0.1 mM Na_2_EDTA, pH 7.2). Lysis reaction was stopped by adding ice-cold complete RPMI medium, cells were washed twice and counted prior use. To generate splenocyte single cell suspensions, spleens were collected and individually placed in 5 mL of RPMI. Then, spleens were transferred in a 100 mm Petri dish and mechanically dissociated into single-cell suspension in RPMI. Red-blood cells were removed by treating spleen cells with ACK, as above. After lysis, cells were washed twice and counted before use.

### Differentiation and Characterization of Bone Marrow-Derived Dendritic Cells Maturation Profile After Sensitization With CPA_160−189_-Loaded p8-PLGA Nanoparticles

DCs were generated from the isolated bone marrow derived cells, as previously described (15). Briefly, cells obtained from the bone marrow were cultured in complete RPMI supplemented with 20 ng/mL recombinant murine GM-CSF (Peprotech, London, UK). On days 3 and 6, loosely adherent cells were harvested and re-suspended in fresh culture medium supplemented with the same dose of GM-CSF. On day 7, non-adherent cells were harvested and assayed for their phenotype as DCs by staining with PE-conjugated anti-mouse CD11c monoclonal antibody (clone HL3, BD Biosciences, Erembodegem, Belgium). Routinely, the percentage of CD11c^+^ cells were >85%. To analyze DC maturation induced by PLGA nanoparticles, DCs were harvested on day 7 of culture, seeded in 24-well plates at a density of 1 × 10^6^ cells/mL and cultured in the presence of (i) p8-PLGA nanoparticles or (ii) CPA_160−189_-loaded p8-PLGA nanoparticles, for 24 h at 37°C in a humidified CO_2_ incubator. DCs in medium alone were used as negative control. At the end of incubation, cells were washed to remove free PLGA nanoparticles followed by a wash with FACS buffer (PBS – 3% (v/v) FBS). The cells were then labeled with R-PE-conjugated anti-mouse CD40 (clone 3/23), CD80 (clone 16-10A1), MHCI (clone SF1-1.1) (all used in 1:100 dilution) or MHCII (clone 2G9; 1:200 dilution) monoclonal antibodies for 30 min at 4°C in the dark. All antibodies were purchased from BD Biosciences. After staining, cells were washed with FACS buffer and subjected to flow cytometric analysis using a FACS Calibur system (Becton-Dickinson, San Jose, CA, USA) running CellQuest software. Data were analyzed using FlowJo software version 10.0 (Tree Star Inc., Ashland, OR, USA).

### Vaccination Schedule and *Leishmania infantum* Challenge of Mice

BALB/c mice (*n* = 25/group) were vaccinated subcutaneously in the upper and dorsal region with CPA_160−189_-loaded p8-PLGA nanoparticles re-suspended in a total volume of 0.1 mL of sterile PBS. Taking into account the CPA_160−189_ loading, each mouse received 2 μg of CPA_160−189_. Two booster doses followed at 2 week intervals. Mice receiving equal amounts of p8-PLGA nanoparticles in PBS in relation to CPA_160−189_-loaded p8-PLGA nanoparticles or PBS alone served as controls. Two weeks after each injection, mice (*n* = 5/group) were euthanized and spleens were collected to analyze the immune responses induced by vaccination. Then, the remaining vaccinated and non-vaccinated mice were challenged by injecting intravenously 2 × 10^7^ stationary phase *L. infantum* promastigotes. Non-vaccinated non-infected (naïve) mice served as negative control group. The prophylactic efficacy of CPA_160−189_-loaded p8-PLGA nanoparticles was assessed in spleen and liver at 4 and 16 weeks post challenge.

### Antigen-Specific Cellular Activity in Spleen

Spleen cells were isolated from vaccinated and non-vaccinated mice at 2 weeks after each vaccination, as well as 4 and 16 weeks post *L. infantum* challenge and were cultured in 96-well round-bottom plates at a density of 2 × 10^5^ cells/200 μL/well in the presence of CPA_160−189_ (5 μg/mL) for 96 h at 37°C and 5% CO_2_. Spleen cells cultured in medium alone or in the presence of Con A (6 μg/mL; Sigma-Aldrich, St. Louis, MO, USA) served as negative or positive control, respectively. Cells proliferation was measured by [^3^H]-TdR (Perkin-Elmer, Boston, MA, USA) incorporation during the last 18 h of the culture period and subsequent measurement of [^3^H]-TdR incorporation was determined on a microplate scintillation β-counter (Microbeta Trilux, Wallac, Turcu, Finland). All assays were performed in triplicates and results were obtained as counts per minute (cpm). Stimulation index (S.I.) was calculated according to the following formula: S.I. = cpm measured in lymphocytes in the presence of antigen or mitogen / cpm measured in lymphocytes in medium alone.

In parallel, similar spleen cell cultures from each mouse group were performed for the determination of IL-2, IFN-γ, TNFα, IL-4, and IL-10 production. Briefly, spleen cells were plated in 24-well plated at a density of 2 × 10^6^ cells/mL and incubated for 72 h in the presence of CPA_160−189_ (5 μg/mL) or SLA (12.5 μg/mL). At the end of incubation, cell-free supernatants were harvested by centrifugation, aliquoted, and stored at −80°C until assayed for cytokine production with magnetic bead multiplex array (Millipore, Billerica, MA, USA). Data were acquired on a Luminex 200 (Oosterhoot, The Netherlands) and analyzed using xPONENT software (Luminex).

### Endogenous CD4^+^ and CD8^+^ T Cell CPA_160−189_-Specific Response

CD4^+^ and CD8^+^ T cell CPA_160−189_-specific activation was measured by using intracellular IFN-γ staining as described previously (14). Briefly, 2 weeks after the end of vaccinations, 1 × 10^6^ spleen cells were cultured in the presence of CPA_160−189_ (5 μg/mL) or medium alone for 48 h at 37°C and 5% CO_2_. At the last 4 h of incubation, cells were exposed to brefeldin A (2.5 μg/mL), washed in FACS buffer and stained with either FITC-conjugated anti-CD4 (clone L3T4) or anti-CD8 (clone 53-6.7) mAbs (1:100 dilution; BD Biosciences) for 30 min. Next, cells were fixed with 2% paraformaldehyde, stained with PE-conjugated anti-IFN-γ (clone XMG1.2) mAb (1:100 dilution; BD Biosciences) in permeabilization buffer for 30 min at 4°C in the dark. After a washing step with FACS buffer, 20,000 cells were analyzed with flow cytometry and data were processed as previously described.

### Evaluation of Parasite Load by Quantitative Polymerase Chain Reaction (qPCR)

The parasite load in spleen and liver was evaluated at 4 and 16 weeks post *L. infantum* challenge by quantitative PCR (qPCR) targeting a 74 bp region of the *L. infantum* arginine transporter AAP3 gene, according to a previously described protocol (16) with some modifications. Briefly, DNA was extracted from about 10 mg of spleen or liver tissue from infected mice using NucleoSpin TissueMN (Macherey-Nagel, Germany) according to the manufacturer's instructions. The quantity and purity of the DNA were determined with the spectrophotometer NanoDrop® 2000 (Thermo Fischer Scientific). Parasite load was determined using a previously described TaqMan-based qPCR assay (10). Quantification was performed using standard curves prepared from DNA extracted from ten-fold serial dilution of *L. infantum* parasites (range 1–1 × 10^5^). Finally, the total number of *L. infantum* parasites per organ was calculated according to the equation below, where V_elution_ corresponds to the volume of eluted DNA, V_DNAtarget_ to the volume of DNA target used per reaction, W_organ_ to the total weight of the organ and W_biospsysample_ to the weight of the biopsy sample subjected to DNA extraction: number of parasites per organ = (number of parasites per reaction × V_elution_ × W_organ_) / (V_DNAtarget_ × W_biospsy sample_).

### RNA Extraction, Labeling, and Affymetrix Expression Array Processing

Total RNA was extracted from sampled spleen tissue that was aseptically excised, snap frozen in liquid nitrogen, and stored at −80°C using Qiazol (Qiagen, Maryland, USA). RNeasy Microarray Tissue kit (Qiagen) was subsequently used for mRNA enrichment following manufacturer's instructions. Isolated total RNA was checked for integrity using the RNA 6000 Nano LabChip kit on the Agilent Bioanalyzer 2100 (Agilent Technologies, Inc., Palo Alto, CA) and concentration using the ND-1000 Nanodrop (Thermo Fisher Scientific, Wilmington, Delaware USA). Only RNA samples with RIN number >7 were used for microarray analysis. Approximately, 300 ng of total RNA were used to generate biotinylated complementary RNA (cRNA) for each group using the GeneChip® WT PLUS Reagent Kit protocol for Whole Transcript (WT) Expression Array, Rev3. A second cycle of cDNA synthesis followed along with a purification step. Then, 6 μg of the single-stranded DNA was fragmented, labeled with the appropriate labeling reagent, and hybridized to GeneChip® Mouse Gene 2.0 ST arrays (Affymetrix, UK). Arrays were scanned on Affymetrix GeneChip® Scanner 3000 at 570 nm. Microarray analysis was performed on 2 biological replicates per group. Images and data were acquired and analyzed using the Affymetrix® GeneChip® Command Console® Software (AGCC). R/Bioconductor was used for initial microarray quality control, normalization, filtering and differential expression analysis. Background correction, quantile normalization, and probe set summarization was performed using the rma function from “oligo” package (17, 18). Probe sets were annotated with Entrez IDs, Ensembl Gene IDs, Gene Symbols and Gene Names using chip-specific annotation package “mogene20sttranscriptcluster.db.” In order to filter probe sets of consistently low intensity, the median of each probe set intensity across all samples was calculated. Probe sets were required to exhibit an intensity higher that the first quartile (Q1) of the median intensities in more than half of the samples to be considered in downstream analyses. Unannotated probe sets were also removed. Package “limma” (19) was employed to perform differential expression (DE) analysis. MIAME compliant raw and processed microarray data have been deposited at GEO under study ID GSE134661. The Database for Annotation, Visualization, and Integrated Discovery (DAVID v6.8; http://david.abcc.ncifcrf.gov/) was used to investigate the functional enrichment of differentially expressed genes. These analyses allowed the identification of Gene Ontology (GO) biological processes (BP) among the enriched lists and a minimum GO level of 4 to exclude too broad terms. Putative protein-protein interactions were identified using STRING (20). Venn diagrams were generated using Venn diagram tool (http://bioinformatics.psb.ugent.be/webtools/Venn/).

### Short Time-Course Analysis of (Non-)vaccinated Samples

A total of 629 annotated probe sets that were significantly different in at least one of the performed comparisons (adj. *p-value*_*ANOVA*_ <0.05) were utilized in the short time-course analysis, conducted with STEM (Short Time-series Expression Miner) (21). Annotated (spot IDs and Gene Symbols) log_2_-transformed expression ratios from 4 and 16 weeks non-vaccinated infected mice against naïve group were used as input in STEM and therefore the “*No normalization/add 0”* setting was employed. “*Minimum Absolute Expression Change”* across any two time points was set to 0.585, signifying 1.5 fold-change. Parameters “*Maximum Number of Model Profiles”* and “*Maximum Unit Change in Model Profiles between Time Points”* were set at the default values (50 and 2, respectively) and “*STEM Clustering Method”* was selected to create candidate temporal profiles (22). The number of expected genes for each candidate profile to have estimated by performing permutations among the available time-points and genes in the true time-point order were assigned to the candidate profiles. Profiles with significantly more genes assigned than expected (Benjamini-Hochberg *p-value* correction) were determined. Enrichment analysis with Gene Ontology (23) was conducted for each model profile using mouse annotation from the Gene Ontology Consortium (www.geneontology.org/ontology/gene_ontology.obo, GOC Validation Date: 11/17/2017). GO enrichment analysis was constrained to Biological Processes and a minimum GO level of 4 to exclude too broad terms. GO categories with adj. *p* < 0.05 were considered significant. *P-value* correction in GO enrichment was performed by randomization: 500 samples of random genes of equal size as each set being analyzed were drawn and the proportion of random samples that enriched any category with a *p-value* smaller than the true *p-value* was estimated.

### Validation of Gene Expression by Real-Time Quantitative PCR (RT-qPCR)

To confirm observations from the gene chip data, TaqMan® Array 96-Well FAST plate (Applied Biosystems, CA, USA) was used to detect genes (*Cxcl9, Cxcl11, Il21, Lag3, Cd274, Pdcd1lg2, Lilrb4*, and *Lgals9*) in 3 biological replicate RNA samples from each mouse group at 4 and 16 weeks post challenge maintained at −80°C until used. One microgram of RNA were used for cDNA synthesis using the PrimeScript® RT Reagent kit (Takara, Japan). Gene expression was determined by measuring the fluorescence signals in real time with the use of ViiA™7 Real Time PCR System (Applied Biosystems) and critical threshold values were generated using the ViiA™7 Software Version 1.2.1 (Applied Biosystems). HPRT was used as an internal control to calculate fold changes in target genes in spleen biopsy samples obtained from infected mice that have been previously vaccinated with CPA_160−189_-loaded p8-PLGA nanoparticles and non-vaccinated infected mice, by use of 2^−ΔΔCT^ method. The correlation between microarray and qRT-PCR results was analyzed by Spearman's Rho test. Significance level was set as *p* < 0.01.

### Statistical Analysis

All results are expressed as mean ± standard deviation (SD). GraphPad Prism version 6.0 software (San Diego, CA, USA) was used for statistical analysis. One-way ANOVA with multiple comparisons Tukey-Kramer post-test or two-way ANOVA with multiple comparisons Bonferroni post-test were performed, when required, in order to assess statistically significant differences among experimental groups. For microarray analysis, limma moderated *t*-tests and one-way ANOVA were used to identify genes that varied significantly between two or more than two groups respectively and Benjamini-Hochberg procedure was utilized to adjust for multiple comparisons. A value of *p* < 0.05 was considered significant for all analyses, unless stated otherwise.

## Results

### *In vitro* Evaluation of DC Maturation After Uptake of CPA_162−189_-Loaded p8-PLGA Nanoparticles

**Figure 1:**
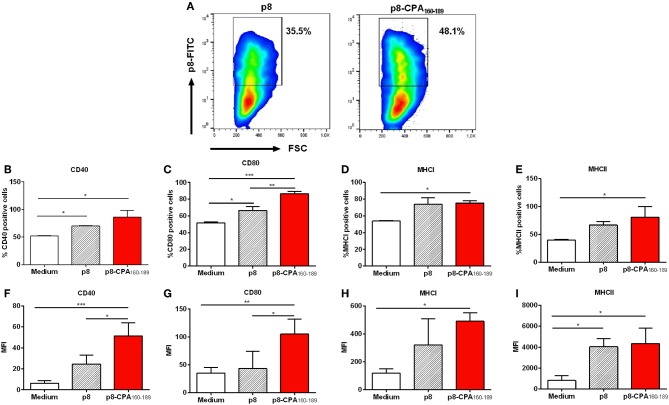
Dendritic cell induced-maturation after CPA_160−189_-loaded p8-PLGA nanoparticles. Bone-marrow-derived DCs were exposed to CPA_160−189_-loaded p8-PLGA nanoparticles for 24 h. DCs cultured in medium alone or p8-PLGA nanoparticles served as controls. Nanoparticle uptake and DC maturation were assessed by flow cytometry. **(A)** Evaluation of CPA_160−189_-loaded p8-PLGA nanoparticle uptake by DCs. Percentage of **(B)** CD40-, **(C)** CD80-, **(D)** MHCI-, and **(E)** MHCII-expressing CD11c^+^ DCs. Mean fluorescent intensity (MFI) of **(F)** CD40, **(G)** CD80, **(H)** MHCI, and **(I)** MHCII on CD11c^+^ DCs. Results represented as mean ± SD of three independent experiments and levels of significance, indicated by *p*-values, were assessed by one-way ANOVA and Tukey's multiple comparison tests (*p < 0.05; **p < 0.01; ***p < 0.001).

We have recently developed PLGA nanoparticles that were surface modified with an 8-mer TNFα-mimicking peptide (will be referred as p8-PLGA nanoparticles) for the specific targeting of TNFRII on DCs' surface. We showed that these modified PLGA nanoparticles were superior to non-modified PLGA nanoparticles in the context of DCs targeting and uptake and induction of protective immune responses against *Leishmania* when encapsulating SLA (10). We proceeded on the construction of peptide-based p8-PLGA nanovaccines through encapsulation of a rationally designed 30-mer peptide, CPA_160−189_, into p8-PLGA nanoparticles. Physicochemical analysis of the synthesized p8-PLGA nanoparticles showed that their characteristics regarding size (417.0 ± 25.1 nm) and charge (−4.9 ± 4.8 mV) could favor their uptake by DCs (Table 1). Their zeta potential was considerably less negative than non-functionalized PLGA nanoparticles, reflecting the successful conjugation between the free anionic carboxyl groups residing on the nanoparticles surface and the amino groups of the p8 peptide. The conjugation efficiency for p8 on PLGA NPs surface was in the range of 63.6 ± 01.5% and caused increase in their average size from 312 ± 3.8 nm to 417 ± 25.1 nm (Table 1). p8 conjugation remained unaffected by CPA_160−189_ encapsulation (Table 1). Specifically, the mean encapsulation efficiency (EE) of CPA_160−189_ was 67.5 ± 12.1%, and the antigen loading was 15 ± 3 μg of CPA_160−189_ per mg of CPA_160−189_ nanoparticles, respectively (Table 1). Investigation of their uptake by DCs with flow cytometry verified that these nanoparticles were efficiently taken up by DCs *in vitro* regardless of whether or not CPA_160−189_ peptide was encapsulated (Figure 1A). As only fully mature DCs are capable of inducing strong and sustained cellular immune responses, the impact of nanoparticle uptake on DC maturation by means of co-stimulatory and MHC molecules expression was then determined. Flow cytometry revealed that DC sensitization with the p8-PLGA nanoparticles triggered significantly higher levels of CD40–, CD80- and MHCI-expressing DCs compared to immature DCs (medium) (CD40: 70.35 ± 0.78% vs. 52.25 ± 0.64%, *p* < 0.05; CD80: 66.65 ± 4.46% vs. 51.45 ± 1.34%, *p* < 0.05; MHCI: 74.15 ± 7.45% vs. 53.80 ± 0.46%, *p* < 0.05) (Figures 1B–D). Even though CPA_160−189_ encapsulation into p8-PLGA nanoparticles did not exhibit any further effect in the number of CD40-, CD80-, MHCI-, and MHCII-expressing DCs compared to DCs sensitized with p8-PLGA nanoparticles (Figures 1B–E), it affected the number of CD40 and CD80 molecules expressed on DCs surface (CD40: 51.35 ± 12.71 vs. 24.63 ± 8.65, *p* < 0.05; CD80: 105.4 ± 26.76 vs. 43.13 ± 30.82, *p* < 0.05), as expressed by MFI, whereas the number of MHCI and MHCII expression remained the same between the two groups (Figures 1F–I).

**Table 1:** Physicochemical properties of the synthesized p8-PLGA nanoparticles^a^.

**Formulation**	**Average size (nm)**	**Zeta potential (mV)**	**Antigen loading (μg/ml)**	**Antigen EE^b^(%)**	**p8 loading (μg/ml)**	**p8 CE^c^ (%)**
PLGA	312.8 ± 3.8	−19.1 ± 5.7	-	-	-	-
p8-PLGA	417.0 ± 25.1	−4.9 ± 4.8	-	-	10 ± 10	63.6 ± 1.5
p8-PLGA-CPA_160−189_	428.4 ± 35.4	−5.7 ± 3.9	15 ± 3	67.5 ± 12.1	9 ± 0.2	61.9 ± 0.1

a *Results are presented as mean ± S.D. (n = 3), where applicable*.

b *EE: encapsulation efficiency*.

c *CE: conjugation efficiency*.

### Vaccination With CPA_162−189_-Loaded p8-PLGA Nanoparticles Led to the Differentiation of CPA_160−189_-Specific T Cell Populations

**Figure 2:**
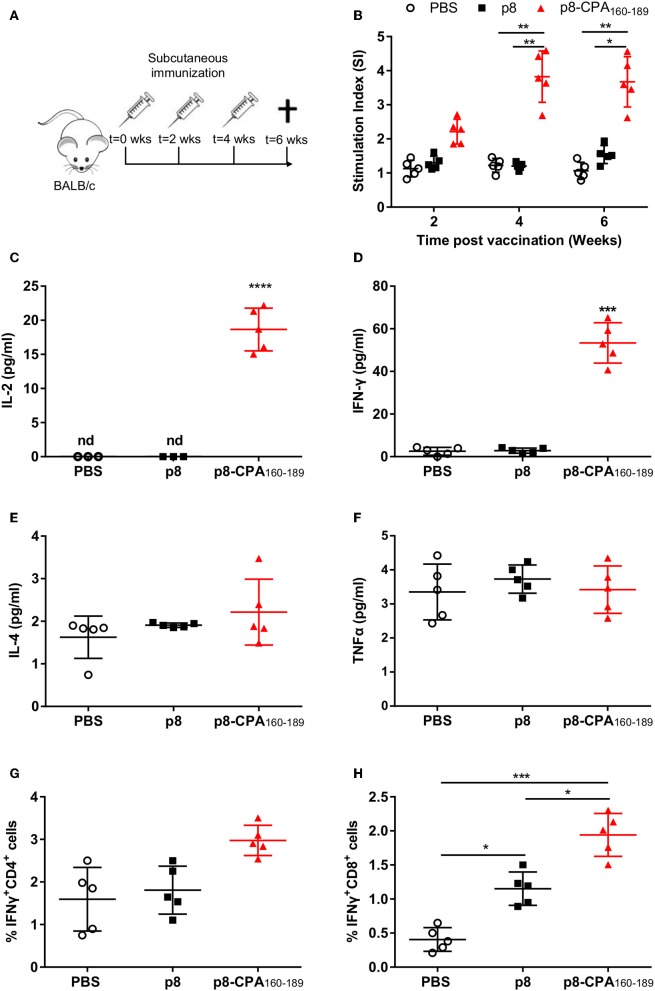
CPA_160−189_-specific immune responses in BALB/c mice post vaccination. **(A)** BALB/c mice were vaccinated thrice with 2 week intervals with CPA_160−189_-loaded p8-PLGA nanoparticles or p8-PLGA nanoparticles. **(B)** Two weeks after each vaccination, spleens were collected and splenocytes were stimulated *in vitro* with CPA_160−189_ for 72 h. CPA_160−189_-specific splenocyte proliferation was determined by [^3^H]-thymidine incorporation after another 18 h of culture. The culture supernatants from spleen cell cultures obtained 2 weeks after the last booster vaccination were assessed for production of **(C)** IL-2, **(D)** IFN-γ, **(E)** IL-4, and **(F)** TNFα by Luminex. Also, spleen cells were cultured in the presence of CPA_160−189_ for 48 h for the assessment of CPA_160−189_-specific **(G)** IFN-γ-producing CD4^+^ T cells and **(H)** IFN-γ-producing CD8^+^ T cells by flow cytometry. Each point depicts the data from a single sample (*n* = 5 per group). Results are shown as mean ± SD and levels of significance, indicated by *p*-values, were assessed by one-way ANOVA and Tukey's multiple comparison tests (*p < 0.05; **p < 0.01; ***p < 0.001). Experiments were conducted twice with similar results and representative results from one experiment are given.

In order to investigate whether vaccination with CPA_162−189_-loaded p8-PLGA nanoparticles induced CPA_160−189_-specific immune responses, BALB/c mice were vaccinated with p8-PLGA nanoparticles having encapsulated CPA_160−189_ or p8-PLGA nanoparticles as a control group, three times at 2 weeks intervals (Figure 2A). As expected, priming with CPA_162−189_-loaded p8-PLGA nanoparticles induced CPA_160−189_-specific responses which were further enhanced after the first booster reaching 3–fold (*p* < 0.01) higher proliferative capacity over the PBS control mice after *ex vivo* stimulation with the CPA_160−189_ (Figure 2B). Importantly, proliferation levels were kept stable after the second booster suggesting the vaccine elicitation of strong immune responses (Figure 2B). The enhanced splenocyte proliferation detected 2 weeks after the second booster in vaccinated animals was complemented by significant levels of IL-2 secretion (18.7 ± 2.6 pg/mL vs. n.d.; *p* < 0.001) in comparison to PBS control group (Figure 2C). In parallel, detection of T_H_1-promoting IFN-γ and TNFα, as well as T_H_2-promoting IL-4 and IL-10 cytokines production showed that these mice developed predominantly a CPA_160−189_-specific IFN-γ-secreting T cell response (52.9 ± 12.3 pg/mL vs. 1.4 ± 0.2 pg/mL; *p* < 0.001) (Figure 2D). In contrast, IL-4 (2.4 ± 0.9 pg/mL vs. 1.8 ± 0.1 pg/mL) and TNFα (3.5 ± 0.9 pg/mL vs. 3.4 ± 1 pg/mL) secretion levels were significantly lower to IFN-γ, and comparable to that of PBS control mice group (Figures 2E,F). Moreover, CPA_160−189_ stimulation did not induce the production of IL-10 from spleen cells obtained from vaccinated mice (data not shown). As the production of IFN-γ provided first indication of the presence of CPA_160−189_-specific CD4^+^ and/or CD8^+^ T cells, their presence was confirmed using ICS. Specifically, an increasing trend that did not reach significance in the percentage of intracellular IFN-γ was detected in CD4^+^ T cells with the higher percentage of intracellular IFN-γ detected within CD8^+^ T cells from mice vaccinated with CPA_160−189_-loaded p8-PLGA nanoparticles as compared to PBS- and p8-PLGA nanoparticle-vaccinated mice (*p* < 0.001 and *p* < 0.05, respectively) (Figures 2G,H). Combined, the above data suggest the induction of CPA_160−189_-specific CD8^+^ T cells after vaccination with the CPA_160−189_ peptide in the context of p8-surface modified PLGA nanoparticles.

### CPA_162−189_-Loaded p8-PLGA Nanoparticles Conferred Early and Transient Protection Against *L. infantum* Challenge in BALB/c Mice

**Figure 3:**
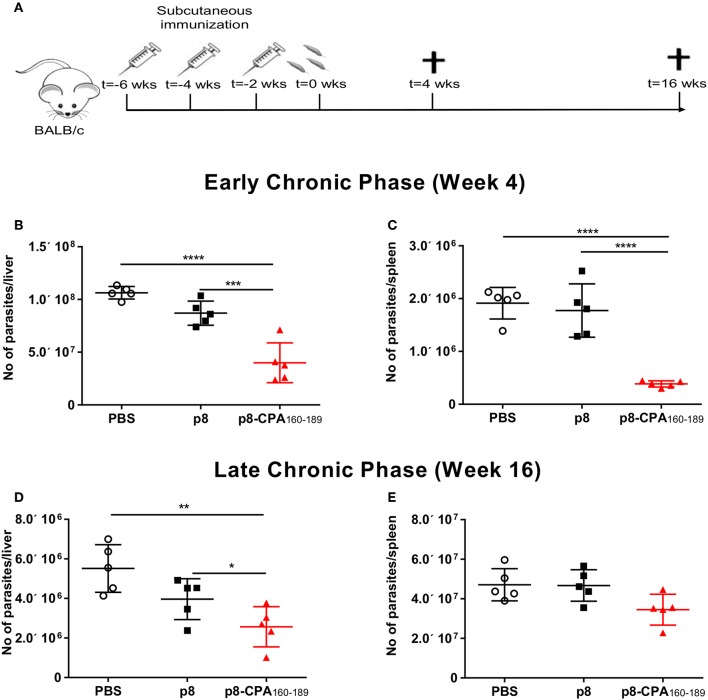
Protective efficacy of CPA_160−189_-loaded p8-PLGA nanoparticles in BALB/c mice against *L*. *infantum*. **(A)** BALB/c mice were vaccinated thrice with 2 week intervals with CPA_160−189_-loaded p8-PLGA nanoparticles or p8-PLGA nanoparticles and then were challenged with *L*. *infantum* promastigotes. Parasite load was assessed at 4 and 16 weeks post challenge in liver **(B,D)** and spleen **(C,E)** of vaccinated and non-vaccinated mice by qPCR. Each point depicts the data from a single animal (*n* = 5 per group). Results are shown as mean ± SD and levels of significance, indicated by *p*-values, were assessed by one-way ANOVA and Tukey's multiple comparison tests (*p < 0.05; **p < 0.01; ***p < 0.001; ****p < 0.0001). Experiments were conducted twice with similar results and representative results from one experiment are given.

BALB/c mice represent a close-to-human model for testing anti-leishmanial vaccine activity. Specifically, in this mouse model, hepatic infection is self-resolving and associated with the development of granulomatous inflammation, whereas splenic parasite load persists accompanied by marked alterations to organ microarchitecture. Based on the above infection profile, determination of anti-leishmanial efficacy of CPA_160−189_-loaded p8-PLGA nanoparticles was conducted at 4 and 16 weeks post parasite challenge, corresponding to the early and late chronic phase of infection (Figure 3A). As illustrated, there was a 69.5% (*p* < 0.001) reduction of the liver parasite burden at 4 weeks post challenge compared to PBS control mice group (Figure 3B). Determination of splenic parasite load revealed that vaccination with CPA_160−189_-loaded p8-PLGA nanoparticles induced significant reduction (80.9%, *p* < 0.001) at 4 weeks (Figure 3C), indicating that the pre-existing vaccine-induced T cell responses aided the clearance of the infection during this time-period. However, at 16 weeks of disease, vaccinated mice failed to sustain the low levels of parasite load, since only 59% and 34.7% reduction in liver and spleen, respectively, were determined (Figures 3D,E). Notably, no effect of the carrier control p8-PLGA nanoparticles on parasite load was seen throughout the study, confirming that the protection seen during acute phase of infection was CPA_160−189_-specific (Figures 3B–E). Thus, the data suggest that CPA_160−189_-loaded p8-PLGA nanoparticles provided early but transient increased protection against *L. infantum* infection.

### Parasite-Induced Immune Responses During Chronic Disease Masked Vaccination-Induced CPA_160−189_-Specific T_H_1 Immune Responses

**Figure 4:**
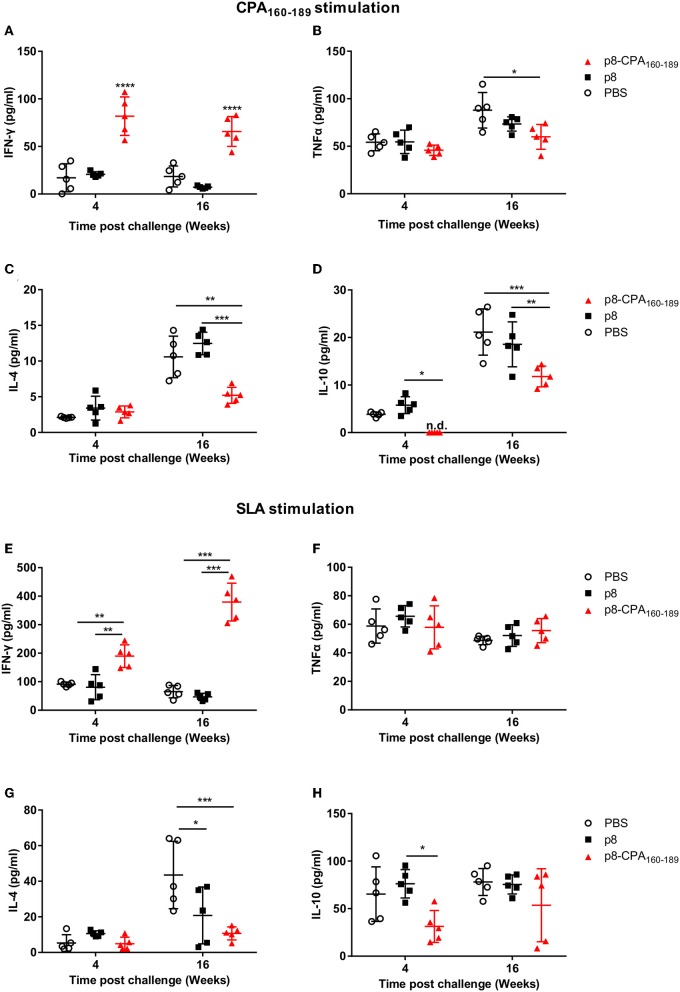
CPA_160−189_-specific and parasite-specific immune responses in vaccinated BALB/c mice 4 and 16 weeks post challenge with *L. infantum*. BALB/c mice were vaccinated thrice with 2 week intervals with CPA_160−189_-loaded p8-PLGA nanoparticles or p8-PLGA nanoparticles and then were challenged with *L*. *infantum* promastigotes. At 4 and 16 weeks post challenge, spleen cells were collected and splenocytes were stimulated *in vitro* with CPA_160−189_ or SLA for 72 h. The culture supernatants were assayed for CPA_160−189_-specific **(A)** IFN-γ, **(B)** TNFα, **(C)** IL-4, and **(D)** IL-10 and parasite (SLA)-specific **(E)** IFN-γ, **(F)** TNFα, **(G)** IL-4, and **(H)** IL-10 production by Luminex. Each point depicts the data from a single sample (*n* = 5 per group). Results are shown as mean ± SD and levels of significance, indicated by *p*-values, were assessed by one-way ANOVA and Tukey's multiple comparison tests (*p < 0.05; **p < 0.01; ***p < 0.001; ****p < 0.0001). Experiments were conducted twice with similar results and representative results from one experiment are given.

To investigate the underlying causes of the discordance between the vaccine-induced parasite control seen in the acute phase and the unpredicted chronic persistence in vaccinated animals, we performed analysis of the CPA_160−189_-specific cellular immune responses in comparison to parasite-induced responses in splenic tissue in both time points (4 and 16 weeks post challenge). Detection of IFN-γ and TNFα, together with IL-4 and IL-10 secretion levels showed that CPA_160−189_-vaccinated mice maintained the same immune response profile as that detected pre-challenge at 4 weeks post-challenge. Specifically, spleen cells retained the high IFN-γ secretion levels (81.9 ± 19.4 pg/mL vs. 15.6 ± 19.2 pg/mL; *p* < 0.001) in comparison to PBS control group (Figure 4A). In contrast, detection of TNFα and IL-4 levels showed that CPA_160−189_-vaccinated mice continued to produce only substantial levels that were comparable to that of PBS control group (TNFα: 45.6 ± 6.9 pg/mL vs. 53.9 ± 11.5 pg/mL; IL-4: 2.7 ± 1.1 pg/mL vs. 2.1 ± 0.3 pg/mL; Figures 4B,C). Furthermore, vaccinated mice continued not to produce IL-10 in response to CPA_160−189_ stimulation (0 ± 0 pg/mL vs. 3.7 ± 0.6 pg/mL; Figure 4D). Notably, the immune response generated in response of spleen cells from CPA_160−189_-vaccinated mice to parasite challenge after *ex vivo* SLA stimulation revealed a similar cytokine profile with that described in the presence of CPA_160−189_. Specifically, it was detected an overall dominance of IFN-γ production (189.7 ± 39.8 pg/mL; Figure 4E) over IL-4 (4.9 ± 3.6 pg/mL; Figure 4F) and IL-10 (31.3 ± 16.9 pg/mL; Figure 4H) in vaccinated mice. Moreover, the levels of IFN-γ were significantly higher than that detected in spleen cells obtained from PBS control mice (189.7 ± 39.8 pg/mL vs. 91.53 ± 7.3 pg/mL, *p* < 0.001) as well as p8-PLGA-vaccinated mice (189.7 ± 39.8 pg/mL vs. 80.8 ± 43.9 pg/mL, *p* < 0.05). On the contrary PBS and p8-PLGA-vaccinated mouse groups produced equivalent and significantly higher amounts of IL-10 compared to vaccinated mice (PBS: 65.3 ± 28.7 pg/mL and p8: 76.1 ± 14.9 pg/mL vs. 31.3± 16.9 pg/mL, *p* < 0.05), indicating an induction of non-protective response against *L*. *infantum*.

It is worth pointing out that during late chronic phase of disease CPA_160−189_-specific cells from vaccinated mice showed the ability to produce not only IFN-γ which continued to be significantly higher in comparison to PBS control (63.5 ± 19.4 vs. 18.5 ±14.3 pg/mL; *p* < 0.001) (Figure 4A), but also elevated levels of the counter-regulatory IL-4 and IL-10 cytokines compared to those detected during acute phase of infection (Figure 4). Importantly, the CPA_160−189_-specific IL-4 and IL-10 secretion levels were 2-fold less in comparison to PBS control group (IL-4: 5.4 ± 1.5 pg/mL vs. 10.8 ± 3.5 pg/mL; *p* < 0.01 and IL-10: 11.7 ± 2.6 pg/mL vs. 20.5 ± 5.9 pg/mL; *p* < 0.01) (Figures 4C,D). Regarding TNFα production levels, these were maintained the same between the two time points in the vaccinated mice group, but they were about 1.5-fold less compared to those detected in PBS control group (57 ± 17.2 pg/mL vs. 90 ± 25.3 pg/mL; *p* < 0.05) (Figure 4B). Assessment of the parasite-specific responses showed that splenic cells from vaccinated mice continued to produce significantly higher amounts of IFN-γ compared to PBS control group in response to SLA stimulation (379.5 ± 66.3 pg/mL vs. 65.2 ± 21.8 pg/mL; *p* < 0.001; Figure 4E). On the other hand, IL-4 and IL-10 production levels were significantly increased over those detected at 4 weeks but they were lower in comparison to PBS group (IL-4: 10.7 ± 3.7 pg/mL vs. 43.5 ± 18.9 pg/mL, *p* < 0.05; IL-10: 53.6 ± 38.3 pg/mL vs. 78 ± 14.2pg/mL; n.s. Figures 4G,H whereas TNFα production remained stable and in the same levels as those detected in PBS control group (55.6 ± 8.5 pg/mL vs. 48.7 ± 3 pg/mL; n.s) (Figure 4F). In all cases, the mice which were vaccinated with p8-PLGA nanoparticles showed the same profile to PBS control group regarding cytokines production (Figure 4), further supporting the lack of protection against parasite challenge.

Overall, these data suggest that the induced cellular responses raised against parasite during chronic phase of disease could mask the expansion of protective immune responses directed to a single peptide, CPA_160−189_, and further enhance the induction of counter-regulatory non-protective immune responses resulting to partial vaccine-protection against infection.

### Microarray Analysis Revealed Distinct Immune Profiles Among Vaccinated and Non-vaccinated During Early Chronic Phase of Infection

**Figure 5:**
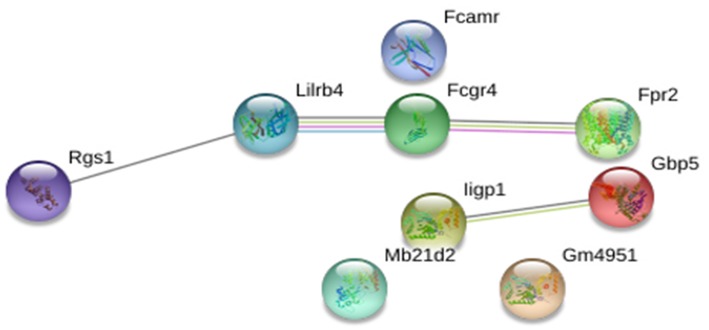
STRING analysis of the 10 DEGs that were significantly up-regulated (*p* < 0.05, fold change>2) in non-vaccinated (PBS) mice at early chronic phase of VL. STRING analysis achieved a PPI network of 9 nodes and 4 edges, with PPI enrichment *p* = 0.000267. Lines in different color represent 7 types of evidence used in predicting associations. Red line: fusion evidence; green line: neighborhood evidence; blue line: co-occurrence evidence; purple line: experimental evidence; yellow line: text mining evidence; light blue line: database evidence and black line: co-expression evidence.

We screened for gene expression patterns using global microarray analysis in spleen tissue obtained at 4 weeks post-challenge from mice vaccinated with CPA_160−189_-loaded p8-PLGA nanoparticles and the PBS group. Naïve (non-infected non-vaccinated) mice were used as the control group to determine gene expression levels. Interestingly, vaccinated mice presented the profile of healthy mice since no differentially expressed genes (DEG) were identified when compared to naive mice (Supplementary Table 1). On the other hand, in non-vaccinated mice 10 DEGs were identified, which were all up-regulated (Supplementary Table 1). Among them were the interferon-inducible genes *Iigp1, Gm4951*, and *Gbp2b*, neutrophil activation and attracting mediators i.e., *Fcgr4, Fpr2*, and *Rgs1*, as well as *Lilrb4* which is involved in immune suppression. Analysis of the aforementioned DEGs in the protein-protein interaction space by employing STRING database, revealed that these DEGs were involved in specific immune processes. Specifically, the term “cellular response to interferon-beta” (GO:0035458, FDR = 0.0096) contained interacting genes ***Iigp1*** and ***Gm4951***, whereas the term “myeloid leukocyte activation” (GO:0002274, FDR = 0.0445) contained ***Fcgr4***, ***Fpr2***, and ***Lilrb4*** genes (Figure 5).

### Disease Establishment Was Characterized by Enrichment of a Neutrophil-Related Signature Irrespective of Vaccination

**Figure 6:**
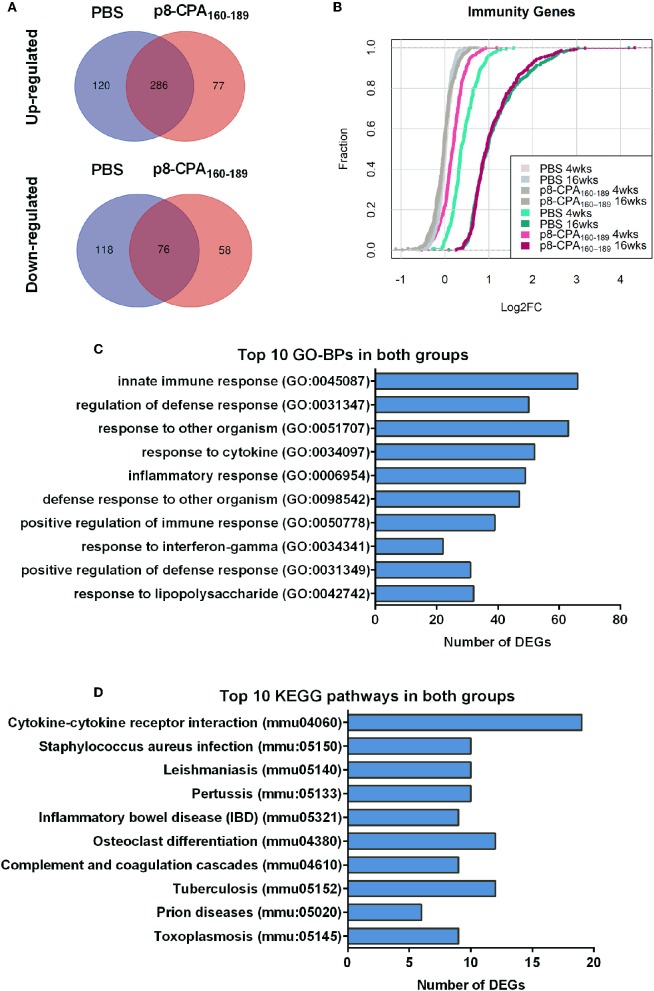
Gene expression profile is similar among vaccinated (p8-CPA_160−189_) and non-vaccinated (PBS) mice at late chronic VL phase. **(A)** Venn diagrams of up- and down-regulated DEGs during infection in vaccinated and non-vaccinated mouse groups. **(B)** Cumulative Distribution Functions (CDFs) of immunity-related genes across contrasts. CDFs in gray where obtained by random sampling of equal numbers from each contrast among all genes. Distribution shifts were assessed using Wilcoxon rank-sum tests. **(C)** Top 10 Gene Ontology Biological Processes (GO-BP) analysis and **(D)** KEGG pathway analysis for the common up-regulated DEGs among non-vaccinated mice and vaccinated mice groups.

During transition to the late chronic phase of infection (16 weeks post challenge) there was significant gene regulation in both groups reflected by the increase in the number of DEGs. Specifically, 620 (410 up-regulated and 210 down-regulated) and 506 (371 up-regulated and 135 down-regulated) DEGs were identified in the spleen compared to naïve mice in PBS and CPA_160−189_-loaded p8-PLGA-vaccinated mice, respectively (Supplementary Table 2). Comparative analysis revealed that ~49% of the DEGs were common in both groups. Specifically, 286 up-regulated and 76 down-regulated genes were found in common (Figure 6A). Wilcoxon rank-sum tests further verified this observation by evaluating the overall shift in Log_2_FC distribution in a selection of immunity-related genes (n = 333) between contrasts. At 4 weeks post challenge the Cumulative Distribution Functions (CDFs) of the non-vaccinated mice were significantly up-regulated relative to the CDFs of the vaccinated mouse group (*p* < 0.05), while they practically overlapped (*p* = 0.72) at 16 weeks post-challenge (Figure 6B).

**Figure 7:**
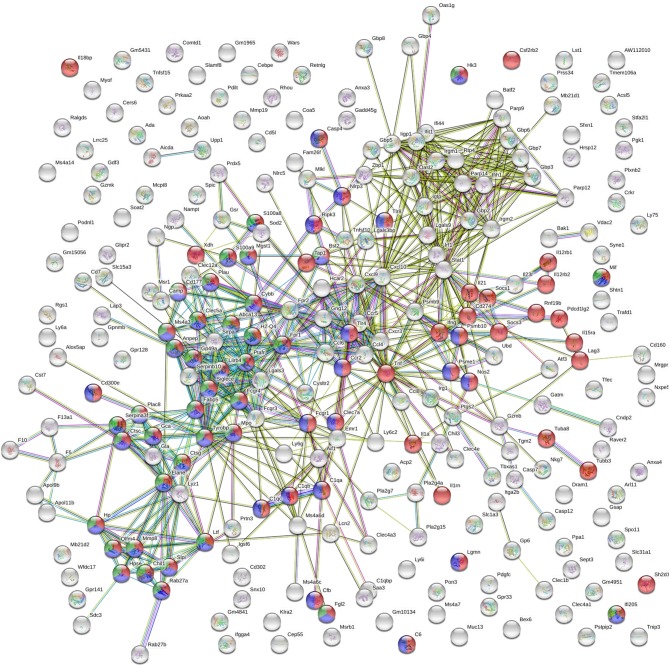
STRING analysis of the DEGs that were significantly up-regulated (*p* < 0.05, fold change > 2) in both vaccinated (p8-CPA_160−189_) and non-vaccinated (PBS) mice at late chronic phase of VL. STRING analysis achieved a PPI network of 271 nodes and 729 edges, with PPI enrichment *p* < 1.0e^−16^. Red shows genes involved in “immune system” term, blue shows genes involved in “innate immune responses” term and green show genes involved in “neutrophil infiltration” term. Lines in different color represent 7 types of evidence used in predicting associations. Red line: fusion evidence; green line: neighborhood evidence; blue line: co-occurrence evidence; purple line: experimental evidence; yellow line: text mining evidence; light blue line: database evidence and black line: co-expression evidence.

GO biological processes and KEGG pathway enrichment analysis of the common up-regulated genes revealed significant (*p* < 0.05 *Benjamini*-corrected) over-representation of terms related to innate and inflammatory responses during the late phase of expression. Specifically, terms such as “innate immune responses,” “inflammatory responses,” “response to cytokine,” “leukocyte migration,” “cytokine-cytokine receptor interaction,” “osteoclast differentiation” and “leishmaniasis” were among the top 10 highest scoring GO-BP terms and KEGG pathways (Figures 6C,D and Supplementary Table 2). Interestingly, the terms related to innate immune responses and defense responses were significantly enriched with genes related to granulocyte infiltration and activation i.e., *S100a8, S100a9, Ptgs2, Elane, Camp, Mpo, Saa3, Fpr1, Fpr2, Slpi*, and *Ctsg*, as well as with T-cell exhaustion molecules, i.e., *Cd160, Lag3, Pdcd1lg2 (Pd-l2), Cd274 (Pd-l1), Lgasl9*, and *Il21*. To investigate the interaction among these common up-regulated DEGs, protein-protein interaction (PPI) network was constructed using STRING. The PPI network consisting of 271 nodes that interacted with 729 edges and an enrichment *p* < 1.0e^−^16, identified a highly significant enrichment for “immune system” (MMU-168256; FDR 1.39E-24), “innate immune system” (MMU-168249; FDR 5.39E-22) and “neutrophil degranulation” (MMU-6798695; FDR 8.81E-18) (Figure 7) further verifying the above analysis.

### Short Time-Series Expression Patterns During Disease Progression

**Figure 8:**
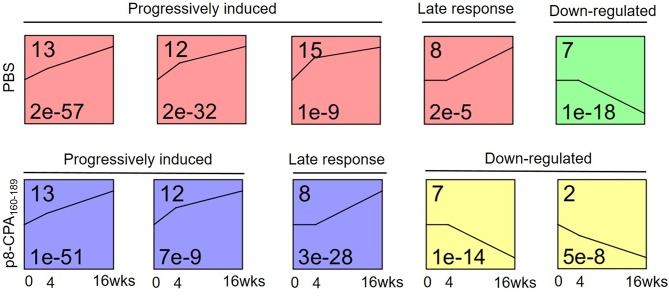
Microarray time course analysis of DEGs. STEM analysis classified 629 DEGs of spleens from non-vaccinated (PBS) and vaccinated (p8-CPA_160−189_) mouse groups at 0 (non-vaccinated non-infected), 4 and 16 weeks into 6 significant patterns of gene expression (*p* < 0.05). DEGs were classified into five main possible profiles according to the temporal gene expression pattern. Each square represents a pattern of gene cluster obtained by the STEM software. The number at the upper left corner of the box represents the profile ID number, and the black broken line in the box represents the overall trend in the expression of all genes in the gene set. The number in the lower left corner indicates the *p*-value assessing the similarity of the gene expression in the cluster.

To investigate gene expression change during progression of the visceral disease, we classified the expression patterns of 628 DEGs from non-vaccinated and vaccinated mice using STEM. We identified a total of 3 significantly enriched patterns of expression among DEGs of non-vaccinated and vaccinated mouse group (*p*-value <0.05, non-parametric clustering algorithm of STEM with Bonferroni correction). Profiles 13, 12, 7 and 8 were common in both mouse groups whereas profiles 15 and 2 only presented enrichment in the non-vaccinated and vaccinated mouse group, respectively (Figure 8). Specifically, profiles 13 and 12 were characterized by progressively induced gene expression pattern over 16 weeks. Profile 8 showed a late response gene expression pattern since their expression started after week 4, whereas profile 7 which showed a down-regulated gene expression pattern starting after week 4. Regarding profile 15 which was exclusive for non-vaccinated mouse group, it showed a progressively induced gene expression. On the contrary, profile 2 which applied only to vaccinated mouse group exhibited a down-regulated gene expression profile. Unexpectedly, few genes were identified common in the identical profiles among the groups.

Subsequently, the DEGs obtained from the different profiles were subjected to GO enrichment analysis. Only profiles 13, 12, and 8 in both mouse groups exhibited significant GO terms enrichment (Table 2). Moreover, comparative GO analysis revealed that the enriched biological processes among DEGs with profile 13 of non-vaccinated mice were analogous to those among DEGs with profile 8 of vaccinated mice i.e., “vesicle membrane,” “killing of cells of other organism,” “extracellular space,” “secretory granule,” “response to oxygen compounds” (Table 2). It must be noted that the terms identified in profile 8 of vaccinated mice were significantly enriched with genes known to be involved in neutrophil infiltration and degranulation, i.e., *Mpo, S100a8, Selp, Slpi, Ctsg*, and *Camp*. As it was mentioned above, gene expression in profile 13 was characterized by progressive induction, with expression changes starting already from week 4, while a later induction, at 16 weeks, was observed for genes in profile 8 (Figure 8). Thus, this earlier gene activation in non-vaccinated as compared to vaccinated mice might be one of the causal factors for the faster disease establishment along with higher parasite loads. Moreover, DEGs in non-vaccinated mice in profile 12, which is characterized by a steeper induction at 4 weeks, and DEGs in vaccinated mice in profile 13 also enriched similar GO-BP terms, namely “cellular response to cytokines” and “response to cytokine” (Table 2). On the other hand, “response to interferon-beta” and “response to interferon-gamma” terms were only significantly enriched in vaccinated mice (Table 2).

**Table 2:** GO-Biological Processes term enrichment of DEGs involved in acute or progressively induced profiles identified by STEM analysis.

		**Term**	**Genes**	***p*-value**	**Corrected *p*-value**
PBS group	Profile 13	GO:0012506~vesicle membrane	10	8.10E-05	0.028
		GO:0044433~cytoplasmic vesicle part	10	8.10E-05	0.028
	Profile 12	GO:0034097~response to cytokine	24	1.20E-05	0.006
		GO:0010033~response to organic substance	35	1.30E-05	0.006
		GO:0071345~cellular response to cytokine stimulus	20	7.00E-05	0.03
p8-CPA_160−189_ group	Profile 13	GO:0035458~cellular response to interferon-beta	13	1.60E-06	<0.001
		GO:0071346~ cellular response to interferon-gamma	16	1.80E-06	<0.001
		GO:0034341~ response to interferon-gamma	17	2.60E-06	<0.001
		GO:0035456~ response to interferon-beta	13	4.70E-06	<0.001
		GO:0034097~ response to cytokine	33	1.80E-05	0.004
		GO:0071345~ cellular response to cytokine stimulus	28	5.00E-05	0.01
	Profile 12	GO:0031640~ killing of cells of other organism	10	2.40E-05	0.016
		GO:0044364~ disruption of cells of other organism	10	2.40E-05	0.016
		GO:0030141~ secretory granule	15	2.60E-05	0.016
		GO:0051883~killing of cells in other organism involved in symbiotic interaction	7	4.50E-05	0.022
		GO:0051818~disruption of cells of other organism involved in symbiotic interaction	7	4.50E-05	0.022
		GO:0005615~ extracellular space	66	5.20E-05	0.022
		GO:0099503~ secretory vesicle	15	5.50E-05	0.022
		GO:1901700~ response to oxygen-containing compound	31	7.40E-05	0.026
	Profile 8	GO:0031347~ regulation of defense response	10	9.20E-05	0.024

### DEGs Related With Stage-Specific Expression Patterns

We next screened for DEGs with specific expression in non-vaccinated mice and vaccinated during the late phase of disease (16 weeks). Comparative analysis of the DEGs among the two groups identified DEGs with either significant up-regulation or suppression (Figure 6A). DAVID functional analysis conducted on the 120 and 77 genes that were significantly up-regulated exclusively in non-vaccinated and vaccinated infected mice did not provide any additional information regarding the immune status among the two groups studied. Specifically, we found that GO functional annotation of the 120 up-regulated genes resulted to only 1 GO-BP term, namely “response to other organism” (Table 3 and Supplementary Table 2). On the other hand, the 77 up-regulated DEGs in non-vaccinated mice were significantly over-represented in GO Biological Processes related not only to “inflammatory responses” and “response to other organism” but also to “regulation of interleukin-10 production” probably related to severity of infection in spleen (Table 3 and Supplementary Table 2). Interestingly, *Tigit* related to CD8^+^ T cell exhaustion was found among the DEGs uniquely expressed in non-vaccinated mice as well as *Il1f9* encoding for the pro-inflammatory cytokine IL-36γ.

**Table 3:** Gene ontology terms enriched by DEGs that were uniquely expressed in non-vaccinated (PBS) and vaccinated (p8-CPA_160−189_) at 16 weeks post *L*. *infantum* challenge.

**Term**	**Genes**	**Adjusted *p*-value**
**PBS group**		
GO:0006954~inflammatory response	18	2.56E-05
GO:0051707~response to other organism	22	4.54E-05
GO:0001819~positive regulation of cytokine production	14	3.17E-05
GO:0031347~regulation of defense response	14	0.005
GO:0098542~defense response to other organism	14	0.005
GO:0050866~negative regulation of cell activation	8	0.006
GO:0050900~leukocyte migration	10	0.006
GO:0032653~regulation of interleukin-10 production	5	0.014
GO:0001818~negative regulation of cytokine production	8	0.018
GO:0032613~interleukin-10 production	5	0.016
**p8-CPA**_**160−189**_ **group**		
GO:0051707~response to other organism	19	0.012

*For the non-vaccinated group, the top 10 GO-BP terms involved are shown*.

### qRT-PCR Validation of Microarray Results

**Figure 9:**
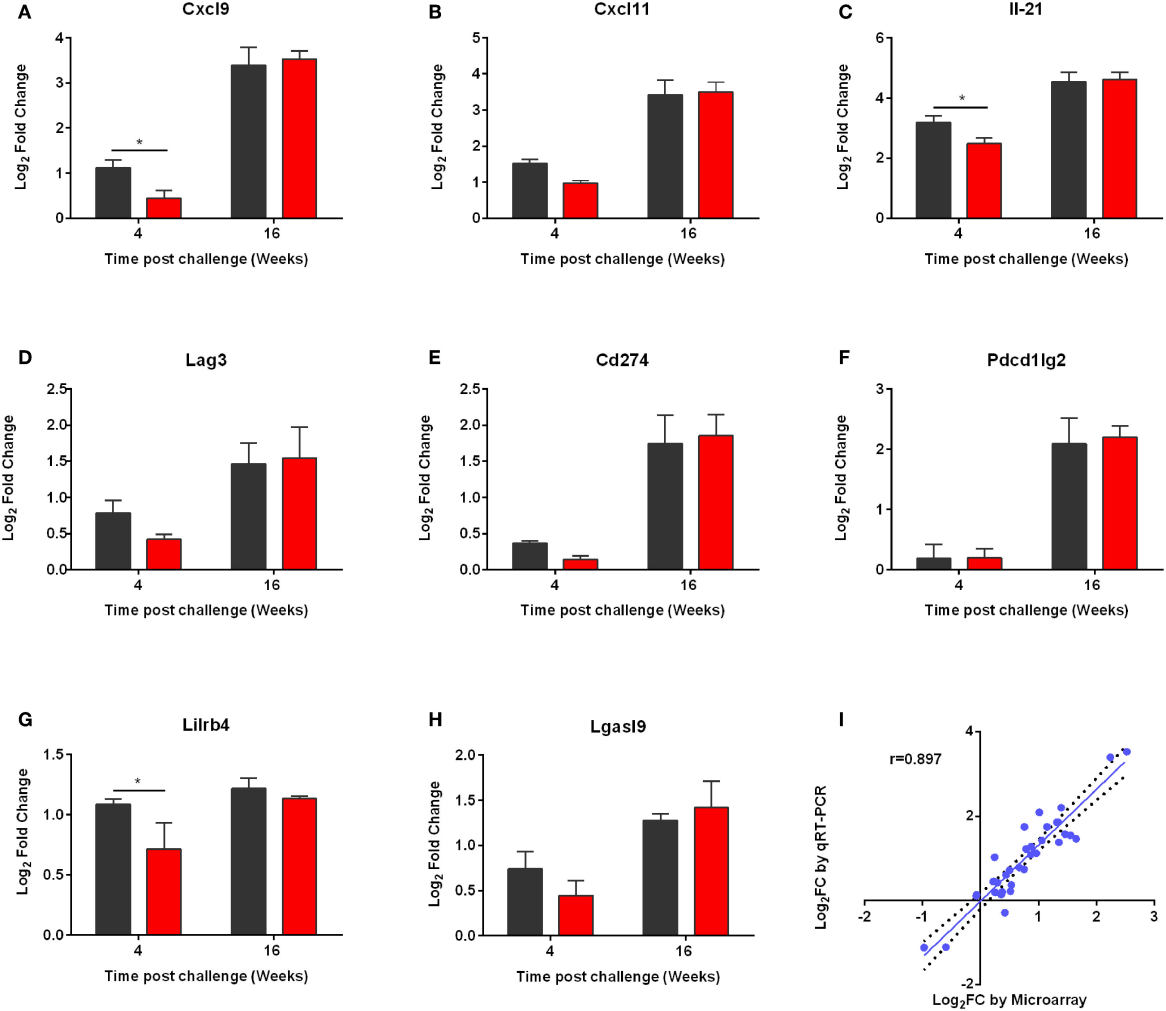
qRT-PCR validation of the microarray data. **(A)** CXCL9, **(B)** CXCL11, **(C)** IL-21, **(D)** LAG3, **(E)** CD274, **(F)** PD-L2, **(G)** LILRB4, and **(H)** LGASL9 were validated. qPCR analysis for the expressions of the above genes was conducted in the RNA samples of non-vaccinated (PBS; black) and vaccinated (p8-CPA_160−189_; red) mice at 4 and 16 weeks post challenge. The data are displayed as Log_2_ fold-change relative to the expression in naïve (non-vaccinated non-infected) mice. Results are presented as means ± SD of three individual mice per group. **(I)** Correlation between microarray and qRT-PCR expression data for 9 selected genes at 4 and 16 weeks post challenge both in vaccinated and non-vaccinated mice. Correlation between microarray and qRT-PCR data was analyzed by Spearman's Rho test. The correlation coefficient was = 0.897, with a statistical significance of *p* < 0.001.

Then, we performed qRT-PCR to ensure that the microarray results could be validated. For this reason, we measured expression of *Cxcl9, Cxcl11, Il21*, as well as *Lag3, Cd274, Pcdc1lg2, Lgasl9*, and *Lilirb4* molecules (Figures 9A–H). The qRT-PCR expression values of all genes confirmed statistically significant differential expression in the same direction as the microarray data with a strong positive correlation (correlation coefficient, *r* = 0.845; *p* < 0.01) (Figure 9I).

## Discussion

In the present study an integrated systems biology approach was applied to investigate the evolving immune response following *L. infantum* challenge in mice vaccinated with CPA_160−189_-loaded p8-PLGA nanoparticles, an experimental vaccine considered capable of inducing sterilizing immunity. Using microarray analysis together with flow cytometry and multiplex immunoassays, we identified transcriptional and cellular profiles that shed light on pathways and gene expression modules associated with the mechanisms that drive progression toward disease.

Visceral leishmaniasis (VL) is a life threating disease and until now there is not any commercially available vaccine for humans (1). Current research efforts on the field are focused in the development of subunit vaccines containing protein fragments or multi-epitope peptides with potentially broad immunological coverage (24). In a previous study, we developed PLGA nanoparticles that were surface-modified with an octapeptide, namely p8, mimicking the TNFα-docking region with TNFRII. These nanoparticles were able to shape the immune response toward T_H_1 and T_H_17 by differentiating DCs into fully functionally DC_1_. Moreover, p8-PLGA nanoparticles having encapsulated SLA were able to confer almost sterile protection against experimental VL through significant induction of IFN-γ-producing CD8^+^ T cells (10). Based on these findings, in the present study we used p8-PLGA nanoparticles to encapsulate a 30-mer peptide, CPA_160−189_, a multi-epitope peptide designed to contain promiscuous overlapping MHC class I- and II-restricted epitopes (9). In accordance with a previous study of ours, p8-PLGA nanoparticles proved suitable carriers for CPA_160−189_, since sensitization of bone-marrow DCs with p8-PLGA nanoparticles induced their maturation as expressed by enhanced number of CD40 and CD80, MHCI and MHCII expressing cells. Moreover, CPA_160−189_ encapsulation further enhance the expression of CD40 and CD80 molecules on DCs surface, which are considered crucial molecules for T cell activation and their differentiation into T_H_1 populations (25, 26). Similarly, we and others have shown that selection of proper carriers for the delivery of various antigenic peptides could serve for the improvement not only of their immunogenicity, since peptides alone cannot induce strong and long-lasting immune responses, but also for guiding antigens to class I or II cytoplasmic major histocompatibility complex loading machinery overcoming the need for classical cross-presentation and facilitating heightened DC stimulation of antigen-specific CD8^+^ T-cells (14, 27, 28).

Indeed, we found that subcutaneous vaccination with CPA_160−189_-loaded p8-PLGA nanoparticles significantly induced the differentiation of antigen-specific CD8^+^ T lymphocytes, minimally induced antigen-specific CD4^+^ T cell populations and presented significantly increased levels of IFN-γ in the spleen. It has been described that CD8^+^ T cell populations are significant for parasite elimination and disease resolution (29–31). Moreover, CD8^+^ memory T cells are responsible for secondary infection control along with CD4^+^ T cells (32). Indeed, mice vaccinated with CPA_160−189_-loaded p8-PLGA nanoparticles were able to control infection with *L. infantum* since ~80 and 70% reduction of parasite loads was detected in spleen and liver, respectively, 4 weeks post challenge compared to the control mouse group. The elevated protection levels were accompanied by increased levels of the pro-inflammatory cytokine IFN-γ. However, as disease progressed, parasite load became similar to that of control mice (16 weeks after infection). These findings suggest that CPA_160−189_-loaded p8-PLGA nanoparticles may have induced preferentially terminally different T cells, since effective vaccination has been shown to be constituted by multi-functional CD4^+^ and CD8^+^ T cells able to expand in the presence of antigenic stimulus and eventually control the disease (33). Also, these mouse group did not produce increased amounts of TNFα in comparison to non-vaccinated mice, further supporting the reduced protection levels against *L. infantum* infection. TNFα is known to induce - alone or in synergism with IFN-γ – the production of NO and ROS by macrophages resulting in parasite elimination (34). Thus, absence of specific induction of TNFα against parasite nicely correlates with the increased parasite load detected at late-chronic phase of disease. Moreover, vaccinated mice acquired the ability to produce IL-10 in response to antigenic stimulus, even though at lower levels than IFN-γ. In VL experimental model, IL-10 plays a significant role in disease establishment by suppressing CD4^+^ and CD8^+^ T cell activation; high levels of IL-10 have been observed to induce CD8^+^ T cell exhaustion and dysfunction (35).

In order to identify the effect of *L. infantum* infection on expression of immune-related genes over the course of infection and eventually on vaccine efficacy, transcriptome analysis in splenic tissue of vaccinated and non-vaccinated mice was conducted at 4 and 16 weeks post challenge. As expected, expression data from non-vaccinated infected mice were significantly different from those in vaccinated mice at 4 weeks of infection reflecting differences in the parasite load related to the elevated protection levels. At this time point, vaccinated mice did not display any alteration regarding gene expression when compared to naïve mice, indicating their healthy profile further depicted by the significantly reduced levels of parasite load in spleen. On the other hand, non-vaccinated mice despite the fact that exhibited a relative low number of DEGs displayed positive regulations of pathways and gene modules related to type I interferon signaling at 4 weeks post challenge, suggesting that IFN-α/β might play important role in infection with *L. infantum*. Dominance of interferon-related pathways was also seen in a transcriptomics study conducted in the hamster model of VL, with about 30% of all genes scored as interferon inducible (36). Blood transcriptomics conducted in asymptomatic infected individuals with *L. infantum* as well as in active VL patients, also revealed enhancement of type I interferon pathway in both cases. The above research group suggested that the response induced by IFN-α/β signaling might have a dual role on the context and clinical status induced by *L. infantum* (37). Specifically, a fine regulation of IFN-α/β expression and of the transcriptional programs induced by those cytokines could promote a protective response in asymptomatic individuals. On the contrary, an unbalanced signaling by these cytokines during chronic VL could play a similar pathological role as that observed with *Mycobacterium tuberculosis* and *Plasmodium* infections (38, 39). In the case of *Mycobacterium* infection, it was shown that over-activation of IFN-γ and type IFN-α/β signaling pathways promoted hyperactivation of neutrophils, thus supporting a role for them in the pathogenesis in tuberculosis (38). In accordance with those findings, we were also able to detect significant enrichment of neutrophil-related genes from 4 weeks post challenge, including genes implicated in neutrophils infiltration and activation that showed an increasing pattern over-time in parallel with others genes implicated in IFN-related pathways as evidenced by STEM analysis with profiles 13 and 12, respectively.

The interplay of type I interferon signaling and neutrophils has been supported by Martinelli et al. that showed that these signaling pathways are crucial for neutrophils to facilitate some of their own functions (40). In the case of sever systemic infections there is increased concentration of pro-inflammatory cytokines, including type I and II interferons, due to inflammation that results in emergency myelopoiesis and enhanced neutrophil production (41). In BALB/c mice, chronic infection of the spleen with viscerotropic species of *Leishmania* is accompanied by a chronic inflammation resulting to enlargement of the organ, extensive remodeling of the splenic architecture and the late onset of extramedullary myelopoiesis (42). This increased rate of myelopoiesis leads to the induction of myeloid-derived suppressor cells (MDSCs), consisting of monocytic- and polymorphonuclear-MDSCs. It has been shown that MDSC migrate along a gradient of the pro-inflammatory S100A8/9 in cancer models (43, 44). Massive infiltration of neutrophils at the site of *M*. *tuberculosis* infection has been found to result in up-regulated levels of S100A8/9 and MDSC accumulation (45). MDSCs display T-cell inhibitory functions through various mechanisms such as IFN-γ-mediated secretion of NO or ROS, increased arginase activity and PPAR-γ triggering. In accordance we those findings, we detected an increased expression of LILRB4 inhibitory molecule from 4 weeks post challenge in non-vaccinated mice. Importantly, STRING analysis unveiled a direct interaction among LILRB4 and neutrophil activating and chemoattracting factors, i.e., FCGR4 and FPR2, further supporting the infiltration of granulocytic populations with immunosuppressive properties in spleen. This profile was further enriched through the expression of PD-L1, PD-L2, CD160, and TIGIT at 16 weeks post challenge following disease establishment. LILRB4 expression marks tolerogenic DCs and has been reported to be elevated in APCs of cancer patients (46, 47) and decreased in autoimmune diseases (48, 49). Membrane-bound LILRB4 interacts with T cells in a cell-to-cell contact-dependent manner and induces immune suppression or tolerance by inducing anergy in CD4^+^ T cells, suppressing the differentiation of IFN-γ-producing CD8^+^ T cells and inhibiting T cell proliferation and the induction of Tregs and alloantigen-specific CD8^+^ suppressor T cells (50, 51). Moreover, a soluble form of LILRB4 resulting from alternative splicing has been described to interact with T cells and induce anergy (52). Regarding infectious diseases, *Salmonella* infection sufficiently enhances LILRB4 expression on DCs and monocytes and its activation results in increased levels of IL-10 production (53). Analogous profiles have been detected in chronically infected hamster spleen tissues which presented increased mRNA expression of PD-1, CTLA-4, PD-L1, and PD-L2 molecules (54), as well as in dogs with chronic VL having increased expression of exhaustion markers in CD4 T cells (55). The existence of exhausted T cells was also verified in our model by up-regulated levels of IL-21 mRNA from week 4 post-challenge that was significantly increased at 16 weeks. It has been proposed that IL-21 acts as a VL-promoting cytokine through the regulation and expansion of pathogenic anti-inflammatory cytokine IL-10, as evidenced by elevated IL-21 mRNA in splenic aspirate cells from patients with active disease (56), as well as in experimental models of BALB/c mice and hamsters infected with *L*. *donovani* parasite (57, 58). IL-10 is the main disease promoting cytokine in VL. Therefore, IL-10 together with IL21 could participate in disease progression and pathogenesis.

Despite the fact that vaccinated mice did not present this phenotype at 4 weeks post challenge, during transition to late chronic infection there was a significant enrichment of molecules related to type I interferon signaling, neutrophil infiltration, and T cell exhaustion, as depicted in PPI networks and evidenced also by STEM analysis. It has been shown that mostly via their adjuvant action, vaccines often induce not only a neutrophil influx but also an MDSC attraction to the site of application (59–62). MDSC populations are considered essential for protection against exacerbated inflammatory tissue destruction and for shifting immune memory toward a protective T_H_1 response (63). However, they may also interfere with generation of protective memory immune responses through suppression of T cell effector cells during infection. Supportive to this were the findings from depletion studies of MDSC and regulatory T cell populations exhibiting improved clinical outcome and vaccination efficacy (64). Despite the fact that in the present study we did not detect such populations, one cannot bypass the possibility that vaccination with CPA_160−189_-loaded p8-PLGA nanoparticles could have elicited the induction of suppressive populations that were further enhanced during infection after the induction of parasite-specific suppressive populations (65–67).

In the present study, we identified a set of genes indicating an association of enhanced interferon-mediated inflammation, neutrophil infiltration, and T cell exhaustion with *L*. *infantum* infection establishment in spleen of BALB/c mice However, this finding needs further investigation using knock-out mice or depletion studies targeting specific populations i.e., neutrophils or MDSCs in order to shed light to their role during infection. Moreover, it must be stressed out that the murine model of VL requires a high dose of parasites given intravenously—a non-physiological route of infection—in order for the parasite to get established giving only a small piece of knowledge regarding the immune responses during human VL. Supportive to this are the new studies in this area that have shown that saliva components can modify the course of infection when given with low doses of parasites and thus the cell populations involved in the immune response (68–71). Thus, the development of an effective vaccine against *Leishmania* should not only be focused on the selection of leishmanial antigens that are proper for inducing the differentiation of effector T cells but also on the appropriate experimental model for the identification of cell populations having immunomodulatory functions and are elicited during vaccination and/or use of suitable experimental models. In conclusion, in depth research is needed to shed light into the role of these pathways and cell populations in the scopes of designing effective vaccines against leishmaniasis

## Data Availability Statement

The datasets generated for this study can be found in the GEO ID: GSE134661, www.ncbi.nlm.nih.gov/geo/query/acc.cgi?acc=GSE134661.

## Ethics Statement

The maintenance of laboratory mice and *in vivo* studies were performed in SPF (Specific Pathogens Free) conditions at the approved establishments of Department of Animal Models for Biomedical Research, Hellenic Pasteur Institute under the registered codes EL25BIO011, EL25BIO012, and EL25BIO013. Animals were housed at room temperature 22 ± 2°C, relative humidity 40–70% and 12 h light/12 h dark cycle. All procedures complied to PD 56/2013 and European Directive 2010/63/EU, welfare and ethical use of laboratory animals based on 3+1R: Replacement, Reduction, Refinement and Respect and the guidelines of PREPARE (Planning Research and Experimental Procedures on Animals: Recommendations for Excellence), ARRIVEs (Animal Research: Reporting *in vivo* experiments) κ*αι* ARRIGE (Association for Responsible Research and Innovation in Genome Editing). The experimental protocol was reviewed and approved by the Institutional Protocol Evaluation Committee and was licensed under the registered code 4455/10-07-2014, by the Official Veterinary Authorities of Attikis Prefecture. Animals' welfare was assessed by the competent users and was supervised daily by the members of Institutional Welfare Body.

## Author Contributions

MA and EK conceived and designed the experiments. MA, EA, and OK performed the experiments. MA, ST, OK, and EK analyzed the data. MA, AH, CK, and EK contributed with the reagents, materials, and analysis tools. MA, ST, OK, and EK wrote the paper. All the authors read and approved the manuscript.

### Conflict of Interest

The authors declare that the research was conducted in the absence of any commercial or financial relationships that could be construed as a potential conflict of interest.
